# Pyrimidine-2,4-diamine acetone monosolvate

**DOI:** 10.1107/S1600536813001025

**Published:** 2013-01-19

**Authors:** Sergiu Draguta, Bhupinder Sandhu, Victor N. Khrustalev, Marina S. Fonari, Tatiana V. Timofeeva

**Affiliations:** a D. Ghitu Institute of Electronic Engineering and Nanotechnologies, 3/3 Academy Street, MD-2028, Chisinau, Republic of Moldova; b Department of Biology & Chemistry, New Mexico Highlands University, 803 University Avenue, Las Vegas, NM 87701, USA; c X-Ray Structural Centre, A.N. Nesmeyanov Institute of Organoelement Compounds, Russian Academy of Sciences, 28 Vavilov Street, B-334, Moscow 119991, Russian Federation; d Institute of Applied Physics Academy of Science of Moldova, 5 Academy Street, MD-2028, Chisinau, Republic of Moldova.

## Abstract

In the title compound, C_4_H_6_N_4_·C_3_H_6_O, the pyrimidine-2,4-diamine mol­ecule is nearly planar (r.m.s. deviation = 0.005 Å), with the endocyclic angles covering the range 114.36 (10)–126.31 (10)°. In the crystal, N—H⋯N and N—H⋯O hydrogen bonds link the mol­ecules into ribbons along [101], and weak C—H⋯π inter­actions consolidate further the crystal packing.

## Related literature

For the biological activity of pyrimidine derivatives, see: Hall *et al.* (1993 ▶); Gengeliczki *et al.* (2011 ▶). For the crystal structures of related compounds, see: Bertolasi *et al.* (2002 ▶); Draguta *et al.* (2012 ▶). For bond lengths in organic compounds, see: Allen *et al.* (1987 ▶). For hydrogen-bonding graph-set notation, see: Bernstein *et al.* (1995 ▶). 
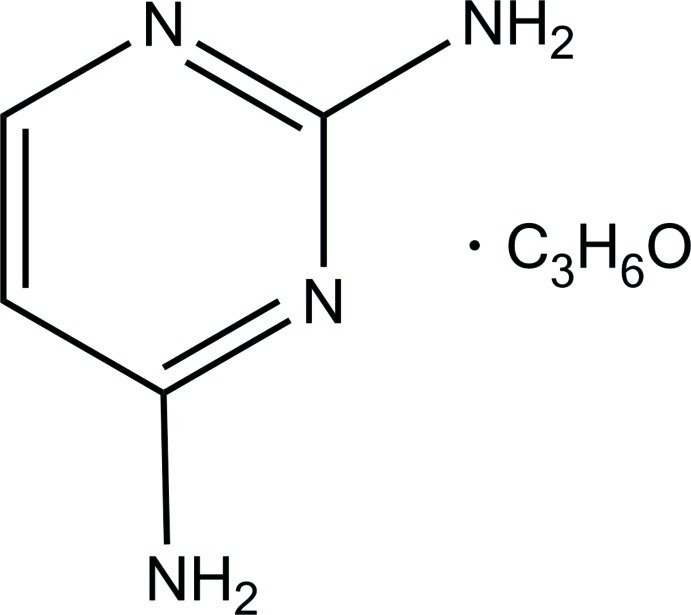


## Experimental

### 

#### Crystal data

C_4_H_6_N_4_·C_3_H_6_O
*M* *_r_* = 168.21
Monoclinic, 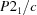

*a* = 8.1594 (15) Å
*b* = 12.728 (2) Å
*c* = 8.7663 (16) Å
β = 99.395 (3)°
*V* = 898.2 (3) Å^3^
*Z* = 4
Mo *K*α radiation
μ = 0.09 mm^−1^
*T* = 296 K
0.30 × 0.25 × 0.20 mm

#### Data collection

Bruker APEXII CCD diffractometer
Absorption correction: multi-scan (*SADABS*; Sheldrick, 2003 ▶) *T*_min_ = 0.974, *T*_max_ = 0.982
9693 measured reflections
2170 independent reflections
1752 reflections with *I* > 2σ(*I*)
*R*_int_ = 0.051

#### Refinement

*R*[*F* ^2^ > 2σ(*F* ^2^)] = 0.048
*wR*(*F* ^2^) = 0.139
*S* = 1.07
2170 reflections
127 parameters
H atoms treated by a mixture of independent and constrained refinement
Δρ_max_ = 0.28 e Å^−3^
Δρ_min_ = −0.28 e Å^−3^

### 

Data collection: *APEX2* (Bruker, 2005 ▶); cell refinement: *SAINT* (Bruker, 2001 ▶); data reduction: *SAINT*; program(s) used to solve structure: *SHELXTL* (Sheldrick, 2008 ▶); program(s) used to refine structure: *SHELXTL*; molecular graphics: *SHELXTL*; software used to prepare material for publication: *SHELXTL*.

## Supplementary Material

Crystal structure: contains datablock(s) global, I. DOI: 10.1107/S1600536813001025/cv5381sup1.cif

Structure factors: contains datablock(s) I. DOI: 10.1107/S1600536813001025/cv5381Isup2.hkl

Supplementary material file. DOI: 10.1107/S1600536813001025/cv5381Isup3.cml

Additional supplementary materials:  crystallographic information; 3D view; checkCIF report

## Figures and Tables

**Table 1:** Hydrogen-bond geometry (Å, °) *Cg* is the centroid of the pyrimidine ring.

*D*—H⋯*A*	*D*—H	H⋯*A*	*D*⋯*A*	*D*—H⋯*A*
N2—H2*A*⋯N1^i^	0.875 (18)	2.191 (18)	3.0608 (18)	177.3 (15)
N2—H2*B*⋯O1	0.871 (16)	2.247 (19)	3.0990 (17)	164.7 (16)
N4—H4*A*⋯O1^ii^	0.879 (17)	2.170 (18)	2.9141 (16)	142.2 (15)
N4—H4*B*⋯N3^ii^	0.900 (18)	2.120 (19)	3.0171 (17)	174.9 (15)
C9—H9*C*⋯*Cg* ^iii^	0.96	2.63	3.5484 (17)	159

Symmetry codes: (i) 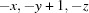; (ii) 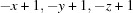; (iii) 
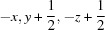
.

**Fig. 1.**
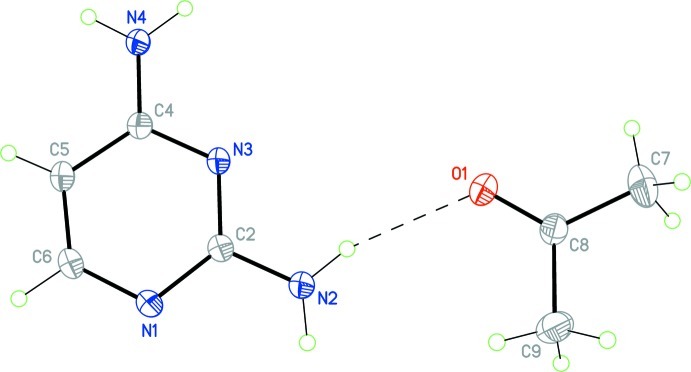
Molecular structure of I. Displacement ellipsoids are shown at the 50% probability level. H atoms are presented as small spheres of arbitrary radius.

**Fig. 2.**
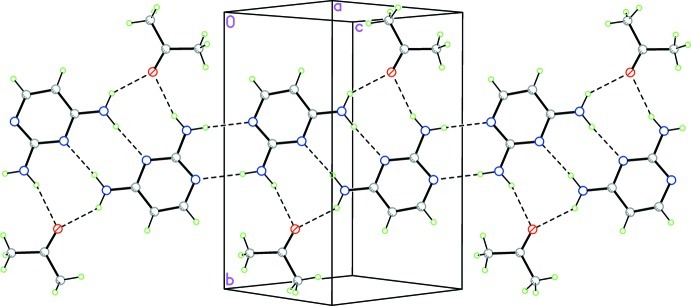
A portion of the crystal packing showing the H-bonded ribbons toward [101]. Dashed lines indicate the intermolecular N—H···N and N—H···O hydrogen bonds.

## supplementary crystallographic information

### Comment

Pyrimidine derivatives are biologically important compounds, because they occur in nature as components of nucleic acids. Pyrimidine-2,4-diamine reacts with 3,4,5-trimethoxybenzyl to form trimethoprim which acts as the nucleic acid inhibitor (Hall *et al.*, 1993) as well as with 3,7-dimethylxanthine to form clusters which represent potential alternate nucleobase pairs, geometrically equivalent to guanine-cytosine (Gengeliczki *et al.* 2011). In the area of drug design and pharmacore mapping, there are several compounds comprising the 2,4-diaminopyrimidinium cations and β- or ζ-diketoenolate anions bound into supramolecular synthons by intermolecular hydrogen bonds (Bertolasi *et al.*, 2002). The presented here crystal structure of the title compound, C_4_H_6_N_4_.C_3_H_6_O, (I) (Figure 1) was determined to study its hydrogen bonding system and to use it in the future for the design of co-crystals with particular properties.

The asymmetric unit of I consists of a pyrimidine-2,4-diamine molecule and an acetone solvate molecule. The pyrimidine-2,4-diamine molecule is planar (r.m.s. = 0.005 Å), with the endocyclic angles covering range of 114.36 (10)–126.31 (10)°. The endocyclic angles at the C2, C4 and C6 carbon atoms adjacent to the N1 and N3 heteroatoms are larger than 120°, and those at the other atoms of the ring are smaller than 120°. The analogous distribution of the endocyclic angles was recently observed by us within the related pyridine-2,5-diamine (Draguta *et al.*, 2012). The bond lengths have the usual values (Allen *et al.*, 1987).

In the crystal, each pyrimidine molecule is connected to two others ones by the intermolecular N2—H2A···N1^i^ and N4—H4B···N3^ii^ hydrogen bonds (centrosymmetric *R*_2_^2^ (8) ring motifs (Bernstein *et al.*, 1995); Table 1), forming the infinite ribbons toward [101] (Figure 2). The acetone molecules are bonded to the ribbons as pendant molecules *via* two intermolecular N2—H2B···O1 and N4—H4A···O1^ii^ hydrogen bonds (Table 1, Figure 2), and are almost coplanar to the ribbon planes (the dihedral angle between the 2,4-pyrimidine and acetone molecules is 7.33 (2)°). The ribbons are packed into layers parallel to (1 0 1), with the interlayer distance of 3.8827 (16) Å). The layers are linked to each other by the intermolecular C9—H9C···π (pyrimidine ring) interactions (Table 1).

### Experimental

The compound I was obtained commercially (Aldrich) as a fine-crystalline powder. Crystals suitable for the X-ray diffraction study were grown by slow evaporation from acetone solution. The crystals of I are very sensitive to air and moisture. Therefore, to keep the quality of the crystal during the experiment, the crystal of I was mounted in vaseline oil.

### Refinement

The hydrogen atoms of the amino groups were localized in the difference Fourier maps and refined isotropically. The other hydrogen atoms were placed in the calculated positions with C—H = 0.93 Å (CH-groups) and 0.96 Å (CH_3_-groups) and refined in the riding model with fixed isotropic displacement parameters [*U*_iso_(H) = 1.5*U*_eq_(C) for the CH_3_-groups and 1.2*U*_eq_(C) for the CH-groups].

### Crystal data

**Table:**

C_4_H_6_N_4_·C_3_H_6_O	*F*(000) = 360
*M**_r_* = 168.21	*D*_x_ = 1.244 Mg m^−^^3^
Monoclinic, *P*2_1_/*c*	Mo *K*α radiation, λ = 0.71073 Å
Hall symbol: -P 2ybc	Cell parameters from 8851 reflections
*a* = 8.1594 (15) Å	θ = 2.3–32.1°
*b* = 12.728 (2) Å	µ = 0.09 mm^−^^1^
*c* = 8.7663 (16) Å	*T* = 296 K
β = 99.395 (3)°	Prism, colourless
*V* = 898.2 (3) Å^3^	0.30 × 0.25 × 0.20 mm
*Z* = 4	

### Data collection

**Table:**

Bruker APEXII CCD diffractometer	2170 independent reflections
Radiation source: fine-focus sealed tube	1752 reflections with *I* > 2σ(*I*)
Graphite monochromator	*R*_int_ = 0.051
φ and ω scans	θ_max_ = 28.0°, θ_min_ = 2.5°
Absorption correction: multi-scan (*SADABS*; Sheldrick, 2003)	*h* = −10→10
*T*_min_ = 0.974, *T*_max_ = 0.982	*k* = −16→16
9693 measured reflections	*l* = −11→11

### Refinement

**Table:**

Refinement on *F*^2^	Primary atom site location: structure-invariant direct methods
Least-squares matrix: full	Secondary atom site location: difference Fourier map
*R*[*F*^2^ > 2σ(*F*^2^)] = 0.048	Hydrogen site location: difference Fourier map
*wR*(*F*^2^) = 0.139	H atoms treated by a mixture of independent and constrained refinement
*S* = 1.07	*w* = 1/[σ^2^(*F*_o_^2^) + (0.0884*P*)^2^] where *P* = (*F*_o_^2^ + 2*F*_c_^2^)/3
2170 reflections	(Δ/σ)_max_ < 0.001
127 parameters	Δρ_max_ = 0.28 e Å^−^^3^
0 restraints	Δρ_min_ = −0.28 e Å^−^^3^

### Special details

**Table:**

Geometry. All esds (except the esd in the dihedral angle between two l.s. planes) are estimated using the full covariance matrix. The cell esds are taken into account individually in the estimation of esds in distances, angles and torsion angles; correlations between esds in cell parameters are only used when they are defined by crystal symmetry. An approximate (isotropic) treatment of cell esds is used for estimating esds involving l.s. planes.
Refinement. Refinement of F^2^ against ALL reflections. The weighted R-factor wR and goodness of fit S are based on F^2^, conventional R-factors R are based on F, with F set to zero for negative F^2^. The threshold expression of F^2^ > 2sigma(F^2^) is used only for calculating R-factors(gt) etc. and is not relevant to the choice of reflections for refinement. R-factors based on F^2^ are statistically about twice as large as those based on F, and R- factors based on ALL data will be even larger.

### Fractional atomic coordinates and isotropic or equivalent isotropic displacement parameters (Å^2^)

**Table:**

	*x*	*y*	*z*	*U*_iso_*/*U*_eq_	
N1	0.12301 (13)	0.39554 (8)	0.11848 (12)	0.0224 (3)	
C2	0.19664 (15)	0.47490 (9)	0.20658 (14)	0.0191 (3)	
N2	0.14277 (14)	0.57309 (9)	0.16744 (13)	0.0255 (3)	
H2A	0.0691 (19)	0.5838 (13)	0.0852 (18)	0.025 (4)*	
H2B	0.197 (2)	0.6249 (13)	0.2183 (19)	0.033 (4)*	
N3	0.31691 (12)	0.46530 (8)	0.33213 (12)	0.0187 (3)	
C4	0.36727 (15)	0.36728 (9)	0.37483 (14)	0.0190 (3)	
N4	0.48650 (14)	0.35701 (8)	0.49903 (13)	0.0240 (3)	
H4A	0.5237 (19)	0.2944 (14)	0.5294 (18)	0.033 (4)*	
H4B	0.539 (2)	0.4120 (14)	0.5498 (19)	0.031 (4)*	
C5	0.29627 (16)	0.27903 (10)	0.29098 (15)	0.0245 (3)	
H5	0.3283	0.2109	0.3202	0.029*	
C6	0.17840 (16)	0.29891 (10)	0.16502 (15)	0.0248 (3)	
H6	0.1329	0.2417	0.1070	0.030*	
O1	0.28515 (12)	0.78374 (7)	0.30884 (11)	0.0285 (3)	
C7	0.30086 (19)	0.97014 (11)	0.31631 (19)	0.0333 (4)	
H7A	0.3699	0.9583	0.4144	0.050*	
H7B	0.2067	1.0120	0.3308	0.050*	
H7C	0.3635	1.0063	0.2489	0.050*	
C8	0.24238 (15)	0.86704 (9)	0.24592 (14)	0.0223 (3)	
C9	0.12660 (17)	0.86932 (11)	0.09351 (16)	0.0297 (3)	
H9A	0.1316	0.8032	0.0418	0.045*	
H9B	0.1592	0.9246	0.0303	0.045*	
H9C	0.0151	0.8816	0.1112	0.045*	

### Atomic displacement parameters (Å^2^)

**Table:**

	*U*^11^	*U*^22^	*U*^33^	*U*^12^	*U*^13^	*U*^23^
N1	0.0249 (6)	0.0168 (5)	0.0226 (5)	−0.0012 (4)	−0.0046 (4)	−0.0021 (4)
C2	0.0203 (6)	0.0162 (6)	0.0199 (6)	−0.0007 (4)	0.0003 (5)	−0.0009 (4)
N2	0.0304 (6)	0.0153 (6)	0.0260 (6)	−0.0001 (4)	−0.0093 (5)	0.0003 (4)
N3	0.0209 (5)	0.0125 (5)	0.0212 (5)	−0.0003 (4)	−0.0012 (4)	0.0001 (4)
C4	0.0190 (6)	0.0155 (6)	0.0217 (6)	−0.0001 (4)	0.0006 (4)	0.0003 (4)
N4	0.0255 (6)	0.0134 (5)	0.0288 (6)	0.0008 (4)	−0.0080 (4)	0.0012 (4)
C5	0.0270 (7)	0.0138 (6)	0.0303 (7)	0.0007 (5)	−0.0025 (5)	−0.0011 (5)
C6	0.0279 (7)	0.0162 (6)	0.0277 (6)	−0.0012 (5)	−0.0027 (5)	−0.0046 (5)
O1	0.0325 (5)	0.0184 (5)	0.0314 (5)	0.0024 (4)	−0.0042 (4)	0.0037 (4)
C7	0.0376 (8)	0.0210 (7)	0.0424 (8)	−0.0053 (6)	0.0099 (6)	−0.0066 (6)
C8	0.0217 (6)	0.0182 (6)	0.0268 (6)	0.0007 (5)	0.0029 (5)	0.0014 (5)
C9	0.0271 (7)	0.0311 (7)	0.0289 (7)	0.0032 (5)	−0.0017 (5)	0.0059 (5)

### Geometric parameters (Å, º)

**Table:**

N1—C6	1.3503 (16)	C5—H5	0.9300
N1—C2	1.3513 (16)	C6—H6	0.9300
C2—N2	1.3505 (16)	O1—C8	1.2196 (15)
C2—N3	1.3552 (16)	C7—C8	1.4948 (18)
N2—H2A	0.871 (16)	C7—H7A	0.9600
N2—H2B	0.875 (18)	C7—H7B	0.9600
N3—C4	1.3474 (15)	C7—H7C	0.9600
C4—N4	1.3428 (16)	C8—C9	1.5054 (18)
C4—C5	1.4137 (17)	C9—H9A	0.9600
N4—H4A	0.879 (17)	C9—H9B	0.9600
N4—H4B	0.900 (18)	C9—H9C	0.9600
C5—C6	1.3644 (18)		
			
C6—N1—C2	114.36 (11)	N1—C6—H6	117.6
N2—C2—N1	116.78 (11)	C5—C6—H6	117.6
N2—C2—N3	116.88 (11)	C8—C7—H7A	109.5
N1—C2—N3	126.32 (11)	C8—C7—H7B	109.5
C2—N2—H2A	120.5 (11)	H7A—C7—H7B	109.5
C2—N2—H2B	116.9 (11)	C8—C7—H7C	109.5
H2A—N2—H2B	121.8 (15)	H7A—C7—H7C	109.5
C4—N3—C2	117.17 (10)	H7B—C7—H7C	109.5
N4—C4—N3	117.59 (11)	O1—C8—C7	121.87 (12)
N4—C4—C5	121.69 (11)	O1—C8—C9	120.66 (11)
N3—C4—C5	120.71 (11)	C7—C8—C9	117.47 (12)
C4—N4—H4A	120.2 (11)	C8—C9—H9A	109.5
C4—N4—H4B	123.3 (10)	C8—C9—H9B	109.5
H4A—N4—H4B	116.2 (15)	H9A—C9—H9B	109.5
C6—C5—C4	116.64 (12)	C8—C9—H9C	109.5
C6—C5—H5	121.7	H9A—C9—H9C	109.5
C4—C5—H5	121.7	H9B—C9—H9C	109.5
N1—C6—C5	124.78 (11)		
			
C6—N1—C2—N2	−178.34 (11)	C2—N3—C4—C5	−0.04 (18)
C6—N1—C2—N3	0.25 (19)	N4—C4—C5—C6	−178.77 (12)
N2—C2—N3—C4	177.85 (11)	N3—C4—C5—C6	1.21 (19)
N1—C2—N3—C4	−0.75 (19)	C2—N1—C6—C5	1.10 (19)
C2—N3—C4—N4	179.95 (11)	C4—C5—C6—N1	−1.8 (2)

### Hydrogen-bond geometry (Å, º)

Cg is the centroid of the pyrimidine ring.

**Table:**

*D*—H···*A*	*D*—H	H···*A*	*D*···*A*	*D*—H···*A*
N2—H2*A*···N1^i^	0.875 (18)	2.191 (18)	3.0608 (18)	177.3 (15)
N2—H2*B*···O1	0.871 (16)	2.247 (19)	3.0990 (17)	164.7 (16)
N4—H4*A*···O1^ii^	0.879 (17)	2.170 (18)	2.9141 (16)	142.2 (15)
N4—H4*B*···N3^ii^	0.900 (18)	2.120 (19)	3.0171 (17)	174.9 (15)
C9—H9*C*···*Cg*^iii^	0.96	2.63	3.5484 (17)	159

Symmetry codes: (i) −*x*, −*y*+1, −*z*; (ii) −*x*+1, −*y*+1, −*z*+1; (iii) −*x*, *y*+1/2, −*z*+1/2.
